# Quality and Content Analysis: Can YouTube Videos on Agoraphobia Be Considered a Reliable Source?

**DOI:** 10.7759/cureus.43318

**Published:** 2023-08-11

**Authors:** Ayse Erdogan Kaya, Beyza Erdogan Akturk

**Affiliations:** 1 Psychiatry, Hittite University Çorum Training and Research Hospital, Çorum, TUR; 2 Psychiatry, Tarsus State Hospital, Mersin, TUR

**Keywords:** youtube, anxiety, agoraphobia, discern, gqs

## Abstract

Background: YouTube, known as an online video platform, is one of the most popular and dynamic video platforms in the world. it provides access to videos with a variety of content, both in health and many other areas. The fact that it is accessible to everyone and free of charge makes it frequently preferred by individuals. The effectiveness of social media platforms on the thoughts and behaviors of individuals has caused YouTube and other similar platforms to be the subject of health research in recent years. Agoraphobia is an anxiety disorder characterized by an intense fear of feeling trapped somewhere. Agoraphobia is a common type of anxiety disorder in society, and cognitive behavioral and psychopharmacological agents are used in its treatment. Our aim in this research is to examine YouTube^TM^ videos on agoraphobia in terms of reliability and quality.

Methods: The first 50 videos related to agoraphobia were included in the analysis, and the duration (minutes), video content, institutions/individuals who uploaded the video, the time elapsed since uploading, total views, and likes were recorded. In addition, all videos were evaluated on two different scales: Quality Criteria for Consumer Health Information (DISCERN) and the Global Quality Scale (GQS). The obtained data were analyzed statistically.

Results: The mean video duration was 11.4±9.38 minutes, the mean views count was 113299.5±333091, and the mean like count was 8512.76±31429.37. Videos were evaluated in terms of content; 50% included general information, 28% agoraphobia experience, and 22% information about overcoming agoraphobia. The GQS and DISCERN scores were significantly higher in professional videos than in non-professionals (*p*<0.005).

Conclusion: As a result of the evaluation of YouTube^TM^ videos about agoraphobia in terms of quality and content, it has been determined that it is necessary to increase the rate of videos prepared by mental health professionals. Psychiatrists and other healthcare professionals working in the field of anxiety should be encouraged to provide videos with scientific and reliable content.

## Introduction

Agoraphobia is a psychopathology characterized by the appearance of panic-like symptoms in public places where it is difficult to escape or help is not easily accessible; it is more common in women, and the overall prevalence is reported to be about 2% [1]. Therefore, individuals with agoraphobia try to stay away from environments they deem fearful [1]. It generally first appears in young adulthood [2]. A complete consensus has not yet been reached on the traumas, personality traits, or social risk factors, leading to the diagnosis of agoraphobia. On the other hand, parental overprotection, the presence of childhood fears or night terrors, and genetic predisposition are discussed as possible etiological factors [2]. Cognitive-behavioral therapy (CBT) and pharmacotherapy are the commonly used treatment modalities in the treatment of agoraphobia, an anxiety disorder seen in a substantial part of the population [3].

In the digital age we live in, individuals with health problems often use the Internet to find quick information and results. This may have effects on patients, depending on the reliability of the information to be obtained from the Internet. Misleading information obtained from the Internet may cause negative results, such as patients abandoning necessary treatments or resorting to unnecessary and harmful alternative methods. In this context, whether internet data on health problems are a reliable source has gained more importance in recent years. YouTube^TM^, the digital video platform established 18 years ago and with more than 500 videos uploaded per minute today, is known as one of the most visited video-sharing sites [4]. Videos uploaded to YouTube^TM^ at any time, by anyone, for free, make YouTube^TM^ a resource in the field of health as well as in other subjects [4]. The lack of any control mechanism regarding the accuracy and reliability of the content of the uploaded videos, and the possible misinformation, especially in health-related videos, may adversely affect the decisions of individuals on health-related issues. For this reason, especially the quality and reliability analysis of YouTube^TM^ content has been the subject of scientific research in the medical field. Although video content and quality analysis studies have been carried out on many subjects, such as claustrophobia, hypoglycemia, rotator cuff tears, etc., it has been determined that there is no YouTube^TM^ study on agoraphobia in the literature yet [5-7]. Our research aimed to examine YouTube^TM^ videos on agoraphobia in terms of quality and content.

## Materials and methods

Study design and search strategy

Our research was conducted by two psychiatrists, and the videos were evaluated simultaneously in different settings. Our research was a study examining the quality and reliability of YouTube^TM^ videos on agoraphobia. On 07/28/2023, the word "agoraphobia" as the search term in the YouTube^TM^ search box and selecting "relevance" from the filtering feature (excluding repetitive videos, non-English, shorter than two minutes and advertising content) were included in the analysis. Since previous YouTube^TM^ analysis studies also included 50 or 100 videos, the sample size was set to 50 [8, 9].

Data collection and evaluation

Fifty videos were reviewed in detail by two independent psychiatrists; video length (minutes), video content, video uploaders, time elapsed since upload, and number of views were recorded. The average daily views of the videos were obtained by dividing the total number of views by the time elapsed since they were uploaded. In addition, the videos were divided into groups, and comparisons were made according to whether the uploaders were professional or not and whether the content was useful or not. Those with scientifically correct content and no misleading information were included in the useful group. The rest, that is, videos with false or unreliable content, were included in the useless group. Professional videos consisted of videos uploaded by doctors or uploaded by health channels and containing speeches by psychiatry professionals. Non-professional videos included those uploaded by patients, youtubers, or others who are not proficient in the relevant field. Whether the video content is useful or useless/misleading was evaluated subjectively by the psychiatrists who conducted the study. For YouTube^TM^ videos about agoraphobia, they were scored independently by two separate authors using the Quality Criteria for Consumer Health Information (DISCERN) and Global Quality Scale (GQS) questionnaires [5].

DISCERN scoring

The DISCERN scale, developed by Singh et al. [10], consists of a scoring system used to evaluate the reliability of consumer healthcare services. The scale we used in our research is a five-point Likert-type scale. This tool scoring includes five items evaluated on a 5-point Likert scale and examines the purposes, reliability, and bias of information sources. According to the scoring result, above three points represents good, three points average, and below three points weak content reliability [5, 8].

GQS scoring

Developed by Bernard et al. [11], the GQS scale is a tool used to evaluate the quality of videos. It is a Likert-type scale in which video contents are scored from one to five according to their usefulness. Five points represent the highest quality, while one point reflects the lowest quality.

Ethics considerations

Our study was carried out in accordance with the ethical principles of the Declaration of Helsinki, and YouTube^TM^ permission was also waived because the videos are open to everyone and free of charge, and ethical approval was not required.

Statistical analysis

The Statistical Package for Social Sciences (SPSS) version 22.0 (IBM Inc., Armonk, NY, USA) statistical program was used to analyze the obtained data. The normal distribution of the data was evaluated with the Kolmogorov-Smirnov test. Variables were not normally distributed, so continuous variables between the two groups were compared with the Mann-Whitney U test. The Spearman correlation test was used for the GQS and DISCERN scores. The chi-square test was used for categorical variables. The p<0.05 value was accepted as statistically significant.

## Results

The mean length of 50 videos watched and evaluated by two independent psychiatrists was 11.4 minutes. The mean number of daily views of the videos was 82.57, and the mean number of likes was 8512.76. Information on the main features of the videos is presented in Table 1.

**Table 1:** Main features of the videos

Parameter	Mean	± SD
Video length (min)	11.40	9.38
View count	113,299.52	333,091.09
Daily view count	82.57	227.16
Like count	8,512.76	31,429.37
Video contents	n	%
General İnformation	25	50
Experience	14	28
Overcome anxiety	11	22
Uploaders	n	%
Mental health professionals	16	32
Health channels	21	42
Personal/Other	13	26

**Figure 1:**
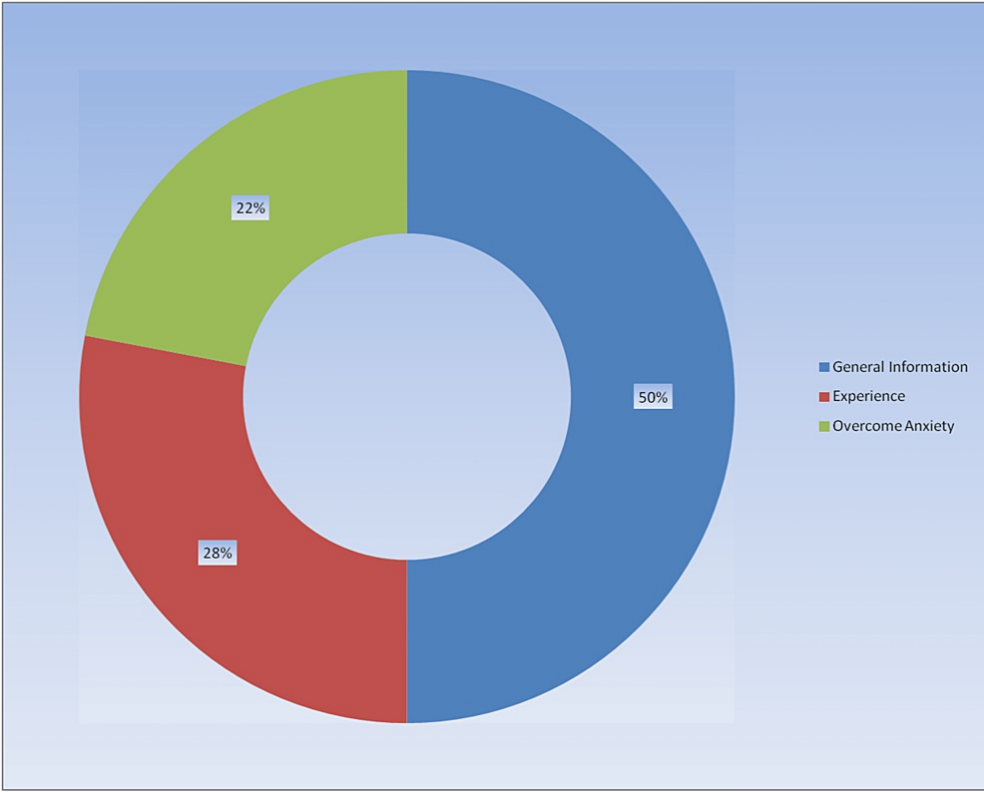
Video contents

When the videos were evaluated as content, 50% contained general information, 28% gave information about experiences, and 22% about overcoming anxiety (Figure 1).

**Figure 2:**
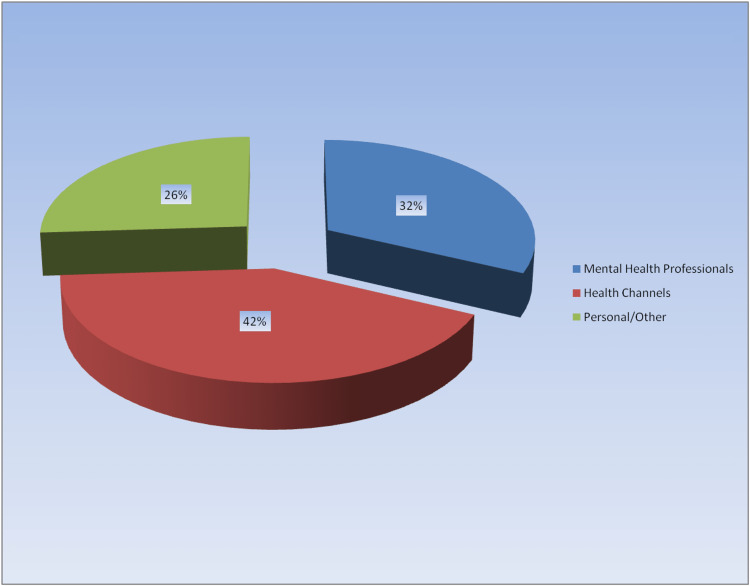
Video uploaders

Specifically, 42% of the videos were uploaded by health channels, 32% by mental health professionals, and the remaining 26% by others (Figure 2).

The match rate between the two psychiatrists regarding the evaluations of the videos was measured by the Cronbach alpha coefficient. The resulting DISCERN and GQS had a significant and positive correlation, and there was strong agreement between the psychiatrists (Table 2).

**Table 2:** Agreement between psychiatrists

	Mean ± SD	p-value	r	Cronbach α
DISCERN 1	3.32±1.00	<0.001	0.875	0.920
DISCERN 2	3.78±0.84			
GQS 1	3.18±1.00	<0.001	0.864	0.906
GQS 2	3.62±0.83			

Since the videos uploaded by health channels and mental health professionals are the videos of individuals who are competent in the field of mental health, these videos were included in the professional group and the others in the non-professional group, and when these two groups were compared, it was determined that, in the professional group, the DISCERN and GQS scores were significantly higher. Other comparisons of these groups appear in Table 3.

**Table 3:** Video features and DISCERN and GQS scores by video uploaders

	Professionals (n:37)	Non-professionals (n:13)	p-value
DISCERN	3.78 ± 0.88	2.89 ± 0.51	0.001
GQS	3.57 ± 0.87	2.92 ± 0.76	0.023
Daily view count	40.59 ± 65.40	202.05 ± 420.68	0.246
Like count	3170.62 ± 13092.58	23717.31 ± 56399.05	0.035
Video length (min)	10.49 ± 9.55	13.99 ± 8.71	0.087

In addition, video contents were divided into two groups by psychiatrists subjectively as useful or useless, and the two groups were compared. The mean number of likes, daily views, and GQS and DISCERN scores were higher in the group including useful videos, and the video durations (minutes) were longer in the useless group (Table 4).

**Table 4:** Video features and DISCERN and GQS scores of useful and useless videos

	Useful (n:39)	Useless (n:11)	p-value
DISCERN	3.76 ± 0.82	2.82 ± 0.75	0.001
GQS	3.63 ± 0.75	2.59 ± 0.86	<0.001
Daily view count	92.83 ± 252.25	46.18 ± 95.78	0.482
Like count	10652.05 ± 35384.20	928.00 ± 1165.06	0.770
Video length (min)	9.14 ± 7.46	19.42 ± 11.36	0.001

## Discussion

According to the main findings of our research, the content of half of the videos was general information, 28% of them were agoraphobia experience, and 22% of them were overcoming agoraphobia. Professional videos had significantly higher GQS and DISCERN scores. The mean video duration was approximately 11.4 minutes, and the average like count was 8,513. About 1/3 of the video uploaders were mental health professionals, 42% were health channels, and the rest were personal/other. Our research is one of the rare YouTube content analysis studies conducted in the field of mental health, and it is noteworthy for mental health professionals in terms of examining the issue of agoraphobia, which is a common disorder in society. With the development of technology and the widespread use of the Internet, digital platforms have become one of the main sources that people use to obtain information on health problems, and approximately 81% of society has started to use the Internet for health research [12]. The YouTube^TM^ video-sharing site is an internet platform that is frequently used to obtain information on any subject and contains videos on various health-related topics. On YouTube^TM^, one of the most preferred video-sharing platforms, there are many videos about general information, experiences, diagnosis, treatment, and methods of coping with diseases [13]. While YouTube^TM^ provides free video content to visitors, it does not guarantee the quality and reliability of the videos. Watched videos may be uploaded by health professionals, as well as daily YouTubers or other people/institutions, and individuals may have difficulty in predicting what kind of content is safe while watching videos. In this respect, it is seen that content analysis studies, which have increased in recent years, are quite popular [14]. It is known that the first research on YouTube content analysis in the medical field was conducted by Keelan et al. on immunization [15]. In the results of this study, it was determined that about half of the videos did not contain reliable information [15]. In this study, we evaluated the content of YouTube^TM^ videos on agoraphobia. According to the results of our research, the mean length of YouTube^TM^ videos on agoraphobia was 11.4 minutes. In other medical studies on YouTube^TM^ content analysis, the duration varies according to the research subject. However, in a study on claustrophobia, the mean video duration was 4.4 minutes [5]; in a study on infertility, it was 8.1 minutes [16]; and in a study on autism, it was 8.53 minutes [17]. The longer mean video durations in our study may be related to the need for a more detailed explanation of specific psychiatric conditions. The duration of the videos may also be related to other reasons, such as the nature of the video uploaders, and the relevant research area.

In our study, the most common video type was videos containing general information about agoraphobia, while the others were experienced and anxiety-coping videos, respectively. In previous Youtube^TM^ content analyses, it has been seen that the content distributions vary according to the fields and topics, but the videos containing general information constitute a significant majority [15, 18-20]. Although the number of daily views and likes of the videos varies according to the popularity of the subject being researched, the number of likes and views of the videos in our research is similar to previous research [7, 21]. In terms of video uploaders, it was determined that health channels and mental health professionals uploaded the most videos on agoraphobia, respectively. The fact that agoraphobia is a common and popular topic may have encouraged healthcare professionals to make videos about it. However, it has been observed that a substantial rate of 26% was uploaded by non-professionals. Although it varies according to the field studied in the literature, it has been observed that the rate of professional videos and non-professional videos is high in some studies [5, 14, 22]. Different results between studies may be due to the prevalence, recognition rate, or characteristics of the diseases in the population. For example, fewer individual/non-professional videos may have been shot on less common and less well-known diseases in the community.

Since the videos included in our study were evaluated by two different psychiatrists; the scores of the scales were correlated with each other. This is a finding that is compatible with other Youtube^TM^ analysis studies [14]. In this study, we evaluated the video contents with the DISCERN and GQS tools. As one of the main findings of our study, both scale scores were significantly higher in the professional video group than in the non-professionals and in the useful group compared to the useless group. This may be related to the fact that the content of the videos uploaded by health professionals and health channels is more adequate and reliable, and these people feel responsible for their profession/institution. In addition, it may be related to the insufficient content of the videos taken by patients, youtubers, or others. The videos uploaded by non-professionals included unscientific methods, and some information was unreliable. Some videos included only individual patient experiences and comments that could not be generalized. Similarly, in a study on YouTube^TM^ videos about contact dermatitis [23], the mean GQS score was reported as 4.5 for professional videos and 2 for non-professionals. Likewise, a different study of YouTube^TM^ videos on dysphagia found significant differences in DISCERN scores between professional and non-professional videos [24]. That is, our findings for DISCERN and GQS scores seem to be in line with other studies in which YouTube^TM^ video content has been analyzed.

Similar to previous studies, in our study, videos were divided into groups as useful and useless, with the subjective classification of two authors [5, 25]. The mean DISCERN and GQS scores of useful videos were significantly higher. This finding is similar to the findings of a YouTube^TM^ analysis study on psoriatic arthritis [25].

In our study, it was observed that non-professional videos had more views and likes than professional videos. The higher duration, likes, and views of non-professional videos may be related to the recognition of video uploaders and the addition of remarkable content. On the other hand, it is an important finding of our research that the mean likes and number of views of the videos did not reflect professionalism, quality, or reliable content. When the literature on YouTube^TM^ content analysis is examined, consistent data on the number of likes and views of professional videos have not been observed, and it has been observed that these numbers vary according to research [5, 26, 27].

Although our research is the first analysis to examine video content on agoraphobia, it has some limitations. These include the fact that only a limited number of videos were included in the study, the videos were evaluated by only two authors and using only two scales, only English videos were evaluated, the videos offer short-term validity because they are content that can be deleted and uploaded instantly, and they do not include the point of view of the patients because they are evaluated only by physicians.

## Conclusions

According to our study, an important part of YouTube^TM^ videos contains general information about agoraphobia. It has been determined that the videos uploaded by non-professionals have lower quality and less broadcast content, and it has been determined that mental health professionals should be encouraged to present useful and reliable videos and to guide patients correctly. Incomplete and incorrect information/directions may be a factor that may impair prognosis and treatment compliance for mentally sensitive individuals. In this respect, YouTube^TM^ videos on health topics should be reviewed by responsible institutions, and videos with incorrect content should be reduced as much as possible. Reliable and healthy information can turn into support for the course of treatment for individuals with mental problems when provided by competent persons and institutions. Although our research contributes to the literature as it is generally compatible with previous YouTube^TM ^content analysis research on medical issues, there is a need for more comprehensive content research.
